# Systematic Review and Meta-Analysis in Chinese Medicine

**DOI:** 10.1155/2014/859309

**Published:** 2014-08-27

**Authors:** William Chi Cho, Myeong Soo Lee, Lixing Lao, Gerhard Litscher

**Affiliations:** ^1^Department of Clinical Oncology, Queen Elizabeth Hospital, Hong Kong; ^2^Korea Institute of Oriental Medicine, Seoul 305-811, Republic of Korea; ^3^University of Hong Kong, Hong Kong; ^4^Research Unit for Complementary and Integrative Laser Medicine, Research Unit of Biomedical Engineering in Anesthesia and Intensive Care Medicine, TCM Research Center, Medical University of Graz, 8036 Graz, Austria

With rich experience accumulating for thousands of years, Chinese medicine (CM) is a treasure for human healthcare. It is a patient-oriented medical system which takes a holistic approach in treating the subjects instead of the diseases [1]. CM possesses a broad spectrum of treatment modalities, including herbal, proprietary CM, acupuncture, moxibustion, and qigong. Selection of therapy is tailor-made on which pattern of disharmony can be identified.

However, the scientific validity of CM due to the lack of scientific evidence is being challenged. Thus, there is a great demand in the knowledge gap to explore the scientific and evidence-based knowledge of CM.

In recent years, both the World Health Organization and the Cochrane Database have documented that CM is effective in the treatment of a number of disorders, especially for chronic diseases. Nevertheless, we need to provide more efficacies and safety evidence to demonstrate its effectiveness in the modern era. Systematic reviews (SR) of high quality randomized controlled trials (RCTs) stand up on the top level of evidence pyramid and are thought to be one of the approaches for evidence-based medicine. With this aim, we invited investigators to submit articles on SR and meta-analysis (MA) that explore the therapy of CM in all aspects.

High standards were applied in the peer-review and selection of manuscripts to be published; out of 35 submitted manuscripts, only five were found to be suitable for publication, which means that the acceptance rate is 14%. Nevertheless, several interesting topics were covered in this special issue.

The efficacy of CM for treating acute mountain sickness (AMS) has been suggested by a large number of published case series and randomized trials, although some trials have demonstrated negative results [2]. Wang et al. reviewed RCTs dealing with CM for treating AMS. A MA showed a beneficial effect in decreasing the score of AMS. However, because of the unclear methodological quality of trials included in their study, a definite conclusion on efficacy and safety associated with CM for AMS cannot be drawn.

Acupuncture for pain control is a well research area [3]. In this special issue, Moon et al. evaluated acupuncture on whiplash-associated disorders (WAD). The authors claimed that this is the first SR on this topic. Among the available six RCTs, four of them suggest that acupuncture has a positive effect on pain in WAD patients. However, none of them showed effectiveness in reducing disability.

Previous evidence reported that CM as an adjunct therapy is uncertain to improve the pulmonary function but may improve clinical symptoms and quality of life (QoL) for chronic obstructive pulmonary diseases (COPD) patients [4]. Chen et al. conducted a SR and MA on this topic, they concluded that current evidence reveals that CM as an adjunct therapy is uncertain to improve the pulmonary function but may improve clinical symptoms and QoL for COPD patients. Studies with large-scale and double-blind RCTs are required to confirm the role of CM in the management of COPD.

In China, the astragalus-containing Chinese herbal prescriptions are quite frequently combined with chemotherapy for lung cancer in clinic [5, 6], yet the area on radiotherapy (RT) is less explored and worth pursuing. He et al. found that, in non-small cell lung cancer treatment, on combining with RT, astragalus-containing Chinese herbal prescriptions may increase the effectiveness and reduce the toxicity of RT. To confirm the exact merit, further rigorously RCTs are warranted.

Besides, Liu et al. analyzed RCTs on CM for postinfectious cough (PIC). They concluded that CM may effectively improve the core symptoms of PIC, act better, and have an earlier antitussive effect, as well as enhancing patients' QoL. In addition, CM is relatively safe and well-tolerated without serious side effects. However, the underlying mechanism of CM in the treatment of PIC is still unclear. Although confirmative conclusions are not obtained, current evidence is encouraging for clinical investigators to pursue further in-depth researches.

On a whole, though some of the studies included in these SRs and MAs were well-designed and comprehensively reported, some limitations still exist. More rigorous, high-quality placebo controlled trials with larger-scale, multicentre for diverse populations are required to provide a high level of evidence.

*William Chi Cho* *William Chi Cho* *Myeong Soo Lee* *Myeong Soo Lee* *Lixing Lao* *Lixing Lao* *Gerhard Litscher* *Gerhard Litscher*
